# P-1540. Treatment Choices for Carbapenem-Resistant Enterobacterales: An Emerging Infections Network Survey

**DOI:** 10.1093/ofid/ofae631.1707

**Published:** 2025-01-29

**Authors:** Samuel E Cincotta, Belinda Ostrowsky, Jessica R Howard-Anderson, Susan E Beekmann, Philip M Polgreen, Maroya S Walters

**Affiliations:** Centers for Disease Control and Prevention, Atlanta, Georgia; Centers for Disease Control & Prevention & Einstein/ Montefiore , New York, NY; Emory University, Decatur, Georgia; University of Iowa, IOWA CITY, Iowa; University of Iowa Carver College of Medicine, Iowa City, IA; Centers for Disease Control and Prevention, Atlanta, Georgia

## Abstract

**Background:**

New antibiotics offer additional treatment options for carbapenem-resistant Enterobacterales (CRE) infections, but data are limited on healthcare provider decision making.

**Figure 1.**
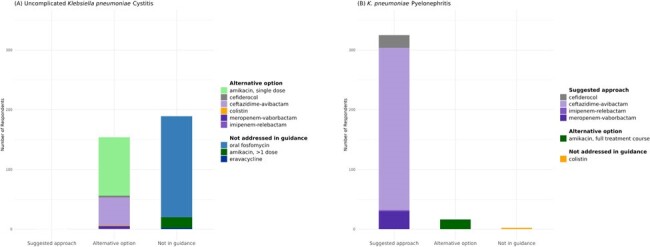
Antibiotic selections for urinary tract infection case scenarios. (A) CASE 1: A 57-year-old female with a history of recurrent urinary tract infections (otherwise healthy with good renal function) presented to the hospital with urinary tract infection symptoms. A urine culture grew Klebsiella pneumoniae resistant to meropenem, ertapenem, and the preferred suggested treatment options for uncomplicated cystitis caused by Carbapenem-Resistant Enterobacterales (nitrofurantoin, trimethoprim-sulfamethoxazole, and ciprofloxacin). (B) The same patient with the same organism and susceptibility testing results as in scenario A presented with pyelonephritis. Note: All antibiotic survey options for urinary tract infection case scenarios are shown in the legend and were selected by at least one respondent.

**Methods:**

We surveyed providers through the Infectious Diseases Society of America (IDSA) Emerging Infections Network (EIN) to understand the current management of CRE infections in the context of new therapeutics. Our survey included four CRE infection scenarios to ascertain antibiotic selection. Selections were classified as suggested, alternative, or not in guidance according to the 2023 IDSA Guidance on the Treatment of Antimicrobial Resistant Gram-Negative Infections. Respondents were asked about barriers to use of selected antibiotics.

**Figure 2.**
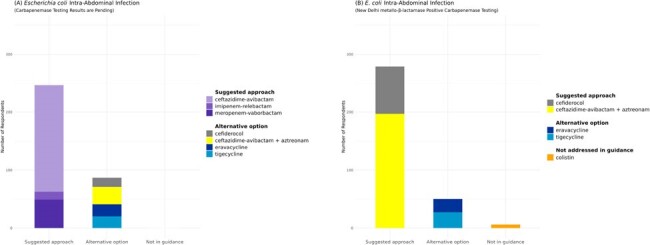
Antibiotic selections for intra-abdominal infection case scenarios. (A) CASE 2: A 64-year-old intensive care unit patient with a new fever has peritonitis and an intra-abdominal abscess. Peritoneal fluid culture grows Escherichia coli resistant to meropenem and ertapenem. Blood cultures are negative and carbapenemase testing is pending. Past medical history does not indicate international travel. No respondent selected the antibiotic survey options of meropenem (extended infusion) plus amikacin or meropenem (extended infusion) plus colistin, both of which are not suggested in the guidance. (B) The lab reports that carbapenemase testing detects the presence of New Delhi metallo-beta-lactamase (NDM) for this isolate. All antibiotic survey options for this scenario are shown in the legend and were selected by at least one respondent.

**Results:**

Overall, 441 (28%) of 1566 EIN members who have ever participated in surveys responded. Among these, 337 (76%) reported treating a CRE infection in the last year. Of the 104 who did not report treating a CRE infection, 93 (89%) did not continue the survey. Among scenarios for treating uncomplicated cystitis with *Klebsiella pneumoniae* resistant to the IDSA suggested agents (**Figure 1**), respondents most often chose an agent not in guidance (187, 55%), primarily oral fosfomycin (169, 49%). For pyelonephritis, most selected suggested agents aligned with IDSA expert opinion (325, 95%), mainly ceftazidime-avibactam (CAZ/AVI; 272, 79%). For treatment of a CRE intra-abdominal infection, 244 respondents (73%) chose CAZ/AVI (a suggested approach) when mechanism testing was pending. When the CRE was confirmed to be New Delhi metallo-beta-lactamase-producing, CAZ/AVI with aztreonam (a suggested approach) was favored (197, 59%) (**Figure 2**). When asked about barriers to use, 57% of respondents indicated there were no barriers for CAZ/AVI, compared to 27% for cefiderocol and 16% for eravacycline (**Table 1**).

**Figure:**
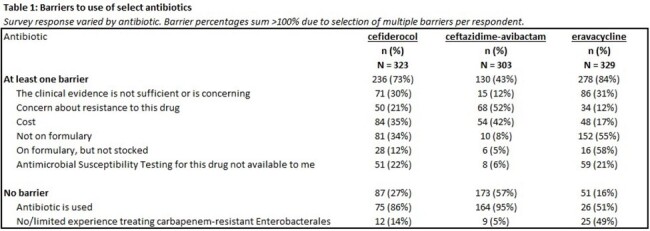

**Conclusion:**

Most clinicians selected newer antibiotics aligned with the IDSA guidance for serious CRE infections but chose an older agent not in the guidance for uncomplicated cystitis, suggesting that treatment selection is complex. The relative preference for CAZ/AVI among similar agents may reflect its longer time in use and that relatively few clinicians reported barriers such as formulary availability and lack of clinical evidence.

**Disclosures:**

**Philip M. Polgreen, MD**, Eli Lily: Advisor/Consultant|Pfizer: Grant/Research Support

